# Consumer Perceptions Influence Supplement Choice: A Narrative Review of Clinically Studied Weight-Management Supplements in Obesity

**DOI:** 10.3390/nu18040702

**Published:** 2026-02-22

**Authors:** Hyeonseok Lee, Jung Hyun Kwak

**Affiliations:** Department of Food Technology and Nutrition, Inje University, Gimhae 50834, Republic of Korea; lhs2616864@naver.com

**Keywords:** dietary supplements, obesity, weight-loss, health-risk behaviors, attitudes, consumer choices

## Abstract

Obesity is a major public health problem with a continuously increasing global prevalence and is associated with various chronic diseases and substantial social and economic burdens. As dietary modification and physical activity alone often have limited effectiveness in achieving sustained weight loss, dietary supplements intended for weight reduction are widely used. However, evidence on the efficacy and safety of these supplements is inconsistent, and consumer use intentions tend to be driven by subjective beliefs and insurance-like perceptions. Accordingly, this study reviewed recent evidence on L-carnitine, green tea extract, glucomannan, and *Garcinia cambogia*, supplements for which weight loss effects have been proposed, to assess their efficacy and safety and to highlight the importance of supplement selection aligned with consumer use contexts. PubMed, MEDLINE, and Google Scholar databases were searched for studies published between 1 January 2020 and 10 October 2025. Although some studies have reported improvements in weight and metabolic indicators, consistent scientific evidence has not yet been established. This review emphasized the need for purpose-driven supplement selection that integrates efficacy, safety, usage context, and evidence level, and the importance of consumers’ critical information appraisal capacity, supported by structured information provision and education.

## 1. Introduction

The World Health Organization defines overweight and obesity as abnormal or excessive fat accumulation that presents health risk to health [1]. Obesity has become a global epidemic, and its severity is progressively increasing [2]. According to 2023 data, approximately 38% of the global population is classified as overweight or obese, and this proportion is projected to reach 51% by 2035 if the current trend continues [3]. The prevalence of obesity in South Korea has steadily increased over the past decade, reaching approximately 38% in 2022, although the prevalence of severe obesity (body mass index [BMI] ≥ 40 kg/m^2^) increased by 2.6-fold when compared with that in 2013 [4]. Obesity occurs when energy intake exceeds total energy expenditure over a prolonged period, resulting in an energy imbalance [5]. This condition induces insulin resistance and hyperinsulinemia, leading to metabolic disorders and markedly increasing the risk of various chronic diseases including type 2 diabetes, cardiovascular disease, multiple types of cancer, and depression. These obesity-related complications extend beyond simple weight gain to systemic metabolic dysfunction, ultimately increasing mortality and morbidity and imposing a substantial burden on healthcare costs from social and economic perspectives [6,7,8,9,10]. Therefore, effective strategies for the prevention and treatment of obesity are recognized by researchers as urgent and important study priorities to mitigate the globally increasing prevalence of obesity, resulting social and economic burdens, and public health challenges. Although caloric restriction and increased physical activity are the core components of traditional weight management, they often fail to achieve clinically meaningful and sustained weight loss when implemented alone. Therefore, dietary supplements that promote weight reduction are widely used as adjunct tools for weight management [11,12]. In South Korea, ingredients that have received individual recognition for weight management functionality include L-carnitine, perilla leaf extract, green coffee bean ethanol extract, and agar (*Gelidium*) extract [13]. Dietary supplements intended for weight management tend to be widely preferred among the general population as components of lifestyle interventions because of their relatively low harm profiles and high accessibility [14]. In the United States, >60% of the population uses dietary supplements, and this proportion continues to increase [15]. Approximately 33.9% of adults attempting to lose weight use dietary supplements [16]. However, based on the evidence to date, consistent scientific evidence regarding the effectiveness and safety of many dietary supplements used for obesity management has not been sufficiently established [17,18]. Meanwhile, in supplementary consumption, consumer perceptions tend to be characterized by a low awareness of the risks associated with inappropriate use, while decisions are often based on intuitive beliefs and personal assessments rather than on scientific evidence. Additionally, supplement use frequently occurs independently of actual health needs, and consumers exhibit inappropriate usage intentions, perceiving supplements as a form of “insurance-like” measure to guard against potential health deterioration [19,20]. Therefore, this study aimed to provide a narrative synthesis of recent literature on four dietary supplements (L-carnitine, green tea extract, glucomannan, and *Garcinia cambogia*) for which weight management and weight loss effects have been proposed in previous studies, with a focus on their effectiveness and safety. Additionally, we emphasized the need for rational supplement selection that integrates intended use in the current supplement market with the level of available evidence. These four supplements were selected because they have accumulated human clinical evidence, including randomized controlled trials, yet continue to show inconsistent findings regarding efficacy and safety. Despite ongoing investigation, they remain widely marketed and consumed, making them clinically and socially relevant for reappraisal in the context of obesity management.

## 2. Materials and Methods

This study was conducted as a narrative review to examine and contextualize available evidence on the efficacy and safety of L-carnitine, green tea extract, glucomannan, and *Garcinia cambogia* in weight management. The literature search was conducted using major electronic databases, including PubMed, MEDLINE, and Google Scholar, targeting studies published in English language between 1 January 2020 and 10 October 2025. Search terms included “L-carnitine,” “green tea,” “green tea extract,” “catechin,” “epigallocatechin gallate (EGCG),” “glucomannan,” “*Garcinia cambogia*,” “weight loss,” “obesity,” “body mass index,” “body fat,” “anti-obesity,” “safety,” “adverse effects,” and “toxicity.” Although this review may be subject to potential researcher bias owing to limitations in the literature selection criteria, it sought to mitigate interpretive bias that could overemphasize the effects in a particular direction by reporting diverse study designs and heterogeneous findings. The outcomes of the included studies were synthesized with a focus on changes in body weight and body composition, metabolic indicators, adverse events, and toxicity-related outcomes to describe the clinical utility, limitations, and current level of evidence for each supplement. The inclusion criteria were as follows: (1) studies conducted in human participants; (2) studies evaluating the effects of supplementation with each supplement on obesity-related metabolic indicators and weight loss; (3) studies assessing the safety of supplementation with each supplement. Eligible study designs were limited to randomized controlled trials (RCTs), prospective observational studies, systematic reviews, and meta-analyses. All included studies are presented in Table 1, Table 2, Table 3 and Table 4, which provide comprehensive information on the authors (years), study design, participant characteristics, intervention duration, supplement formulation, dosage ranges used, primary outcomes (efficacy), and safety and adverse events.

## 3. Effectiveness and Safety of Weight-Management Supplements 

### 3.1. L-Carnitine

#### 3.1.1. Weight Loss Mechanism

L-carnitine is a hydrophilic amino acid derivative of lysine and methionine, and is a key nutrient involved in energy metabolism [43]. Endogenous L-carnitine is primarily synthesized in the liver and kidneys [44], whereas animal-derived foods, such as meat, fish, and milk, are recognized as the main exogenous dietary sources [45]. Based on these metabolic roles, the blood glucose-regulating and lipid-lowering effects of L-carnitine have been proposed as the key mechanisms in obesity management [46,47]. Previous studies have reported that L-carnitine supplementation may be associated with improvements in lipid profiles, lipoprotein (a), and insulin sensitivity, thereby indicating its potential contribution to the alleviation of clinical manifestations of metabolic syndrome and cardiovascular disease [48,49,50]. Additionally, studies involving patients with metabolic syndromes have suggested that L-carnitine may reduce appetite during fasting periods, enhance weight loss through blood glucose stabilization, and attenuate insulin resistance [51]. In general, L-carnitine functions as a primary carrier in fatty acid metabolism and plays an essential role in the β-oxidation of medium- and long-chain fatty acids and in energy production by facilitating the transport of fatty acyl-CoA across the inner mitochondrial membrane into the mitochondrial matrix. In particular, the acetyl-L-carnitine form has important physiological functions that support efficient transport and oxidation of fatty acids [52,53]. Moreover, L-carnitine modulates the expression or activity of regulatory factors involved in lipid catabolism and adipogenesis, including hormone-sensitive lipase, acyl-CoA oxidase, and carnitine palmitoyltransferase I-A, and therefore plays multifaceted regulatory roles across all metabolic processes [54].

#### 3.1.2. Clinical Studies

In a randomized controlled trial (RCT) that analyzed the effects of L-carnitine on fat accumulation and cardiometabolic risk indicators in overweight and obese women with knee osteoarthritis, supplementation with L-carnitine at a dose of 1 g/day for 12 weeks resulted in an improvement in the lipid accumulation product, an index associated with visceral adiposity and insulin resistance [21]. In another RCT that evaluated the effects of L-carnitine supplementation combined with a low-calorie diet on anthropometric measures, supplementation at a dose of 1 g/day for 3 months did not result in a statistically significant reduction in body weight in either the L-carnitine or placebo group. However, a significant reduction in waist circumference (WC) was observed in the L-carnitine group [22]. With respect to anthropometric outcomes (body weight, BMI, WC, and body fat indices), findings across meta-analyses remain inconsistent. According to a comprehensive review by Watanabe et al. [55], although some meta-analyses reported significant weight loss effects, these effects tended to attenuate over time and were more evident in overweight and obese adults. Similarly, Talenezhad et al. [23] reported significant reductions in body weight, BMI, and body fat mass, whereas no significant changes were observed in WC or body fat percentage. A non-linear dose–response analysis indicated the greatest effect at a dose of 2 g/day; however, no consistent dose–response relationship was identified for BMI, WC, or body fat percentage, and effect sizes tended to decline with longer intervention duration [23]. An umbrella meta-analysis also reported significant reductions in body weight, BMI, and WC, particularly in interventions lasting less than 18 weeks and at daily intakes ≥1 g; however, inconsistent findings regarding body weight and BMI were observed across the eight included meta-analyses [24]. Liu [52] concluded that overall weight loss effects were generally limited and suggested that the low bioavailability of L-carnitine may restrict metabolic benefits at conventional intake levels (1–2 g/day), offering an interpretation that contrasts with previous meta-analyses reporting maximal effects at 2 g/day [52]. In contrast to anthropometric outcomes, glycaemic parameters appear somewhat more consistent. A dose–response meta-analysis by Zamani et al. [25] demonstrated that L-carnitine supplementation significantly reduced fasting blood glucose (FBG), glycated hemoglobin (HbA1c), and homeostasis model assessment of insulin resistance (HOMA-IR), with the greatest improvements observed at intakes ≥2 g/day and with an optimal intervention duration of approximately 50 weeks. However, insulin levels did not change significantly in the overall pooled analysis [25]. Taken together, clinical studies have reported statistically significant changes in some metabolic and weight-related outcomes; however, findings remain heterogeneous and are not consistently replicated. Dose–response relationships and long-term durability of effects are not clearly established. Therefore, despite mechanistic plausibility, current evidence does not support firm conclusions regarding clinically meaningful or sustained weight reduction with L-carnitine supplementation.

#### 3.1.3. Safety and Adverse Effects

Overall, serious adverse events have been infrequently reported in clinical studies of L-carnitine supplementation [55]. In the studies included by Zamani et al., predominantly mild gastrointestinal adverse events were reported, with musculoskeletal pain and discomfort observed in some studies [25]. Adverse gastrointestinal and cardiovascular effects, including diarrhea, nausea, abdominal discomfort, dizziness, and palpitations, have been reported following high doses of L-carnitine [23,52]. In some studies, diarrhea-related withdrawals have been reported, and high-dose intake has been associated with increased trimethylamine N-oxide production, suggesting a potential increase in cardiovascular risk [24]. Accordingly, the currently recommended upper safe intake level is 2 g/day, although clinical guidance for this dose remains limited [23]. Based on currently available clinical studies, L-carnitine has generally been well tolerated within the investigated dose ranges and intervention durations. However, these findings do not allow firm conclusions regarding high-dose or long-term use, and safety interpretation should consider dosage, formulation, duration, and study context.

### 3.2. Green Tea

#### 3.2.1. Weight Loss Mechanism

Green tea is made from unfermented *Camellia sinensis* leaves, which preserve the constituents of fresh leaves and results in a high polyphenol content, particularly catechins. A standard cup brewed with 2.5 g of tea leaves provides approximately 240–320 mg of catechins, with epigallocatechin-3-gallate (EGCG) comprising approximately 60–65% of the total catechin content [29,56]. Accumulating evidence suggests that many of the physiological benefits of green tea are primarily attributable to EGCG, the predominant catechin present at high concentrations [57]. Previous studies have shown that green tea confers beneficial effects on various health conditions such as obesity, cancer, hypertension, dyslipidemia, and hyperglycemia [58,59,60,61]. In the context of green tea-induced anti-obesity effects, EGCG has been proposed to function as a pro-oxidant that increases intracellular reactive oxygen species production and activates AMP-activated protein kinases (AMPK). Once activated, AMPK inhibits adipocyte differentiation and lipogenesis and promotes lipolysis and fatty acid oxidation. These actions regulate the expression of key lipid metabolism-related enzymes and transcription factors, thereby reducing adipose tissue accumulation and improving energy homeostasis [62]. In line with AMPK activation, EGCG administration in a high-fat diet-induced obese mouse model significantly attenuated diet-induced weight gain, which was attributed to the decreased adipose tissue mRNA expression of FASN and ACC-1. Reduced FASN mRNA expression is a biochemical marker of inhibited lipogenesis [63]. ACC inhibition enhances fatty acid oxidation and suppress fatty acid synthesis, potentially contributing to a reduction in body fat [64]. EGCG also inhibits bile acid reabsorption and reduces intestinal bile acid levels and lipid absorption, thereby mitigating metabolic abnormalities [65]. Collectively, these findings suggest that EGCG may exert anti-obesity effects and improve lipid metabolism via multiple mechanisms, including inhibition of lipogenesis, enhancement of fatty acid oxidation, and reduced lipid absorption.

#### 3.2.2. Clinical Studies

Overall, evidence suggests that green tea or catechin interventions are associated with improvements in body weight and lipid indices, although the effect magnitude and consistency varied by dose, intervention duration, formulation, and participant characteristics. In a randomized double-blind trial of 45 women with polycystic ovary syndrome, intake of 1.5 g/day of green tea for 3 months significantly reduced body weight, BMI, WC, and hip circumference, while the waist-to-hip ratio (WHR) remained unchanged [26]. In another RCT of patients with type 2 diabetes mellitus, EGCG supplementation at 300 mg/day for 8 weeks significantly reduced body weight, BMI, FBS, and high-sensitivity C-reactive protein levels compared with baseline, although no statistically significant differences were observed when compared with placebo [27]. A dose–response meta-analysis of 26 RCTs demonstrated a clear dose–response relationship for weight loss. Significant reductions in body weight and BMI were observed with intervention durations of ≥12 weeks and doses < 800 mg/day, particularly <500 mg/day. In contrast, WC did not change significantly, which was explained by heterogeneity related to inadequate measurement standardization, inter-observer variability, and possible measurement errors [28]. Similarly, a meta-analysis by Xiao et al. [29] showed reductions in body weight and BMI and improvements in lipid profiles, characterized by increased high-density lipoprotein cholesterol (HDL-C) and decreased low-density lipoprotein cholesterol (LDL-C), triglyceride (TG), and total cholesterol (TC) levels. In contrast, WC, WHR, and several metabolic outcomes, including insulin, glucose, and blood pressure levels, have shown inconsistent findings. HbA1c decreased significantly, particularly at doses < 1 g/day and intervention periods ≥ 3 months. Inter-study heterogeneity has also been noted [29]. In one RCT, the supplementation group exhibited a significant decrease in RQ, indicating reduced carbohydrate oxidation and relatively increased fat oxidation. The CRP and visceral adipose tissue levels were significantly reduced in a supplementation group. HOMA index and total fat mass also decreased significantly, suggesting improved adipose tissue function [31]. A systematic review by Macêdo et al. [30] examining lipid outcomes by Macêdo et al. [30] reported that the effects of EGCG on lipid profiles were condition-dependent. EGCG at 208 mg/day for 12 weeks reduced TC, TG, and LDL levels and increased HDL levels, whereas no changes were observed at 300 mg/day over the same period in pre-menopausal women. Administration of 188.3 mg/day for 20 weeks increased HDL only, and no significant effects were reported with short-term interventions of 4–8 weeks. No lipid improvements were observed with a 97% high-purity EGCG extract, whereas a lower-dose formulation of 54.8 mg/day resulted in reductions in TC, TG, and LDL over the same duration [30]. Overall, green tea or catechin supplementation may be associated with modest reductions in body weight, BMI, and certain lipid parameters under specific doses and intervention durations. However, findings for waist-related indices and several metabolic outcomes remain inconsistent, and results vary by formulation, dosage, and study population. Therefore, while some benefits are suggested, the overall magnitude and consistency of clinical effects are not sufficient to support strong or generalized conclusions.

#### 3.2.3. Safety and Adverse Effects

Across studies evaluating the effects of green tea, the explicit reporting of adverse events is limited. Nonetheless, reviews by Abiri et al. [66] and Brimson et al. [67] suggested that high-dose green tea or catechin intake was associated with potential toxicity, including hepatotoxicity, as well as adverse events such as headache, insomnia, and tachycardia. EGCG was identified as the catechin with the highest hepatotoxic potential, with acute toxicity being dose-dependent, and high concentrations were linked to cytotoxicity mediated by mitochondrial reactive oxygen species generation. A cross-sectional study of 400 pregnant women reported iron-deficiency anemia among those consuming green tea during pregnancy. Dose-dependent adverse gastrointestinal effects were observed with respect to subacute toxicity. In contrast to concentrated extracts, conventional green tea infusions have not consistently been associated with serious adverse events in the reviewed studies. However, safety interpretations should consider formulation, dosage, duration, and individual health status. Intake restriction may be warranted for individuals with renal or hepatic diseases and during pregnancy or lactation to minimize potential adverse effects [66,67].

### 3.3. Glucomannan

#### 3.3.1. Weight Loss Mechanism

Konjac glucomannan (KGM) is a water-soluble dietary fiber derived from *Amorphophallus konjac*, consisting of β-1,4-linked D-mannose and D-glucose monomers. It provides gastrointestinal benefits and has negligible calories [68,69]. KGM has high viscosity and strong water-holding capacity, enabling it to absorb water up to approximately 50 times its own weight. These properties allow the KGM to swell in the gastrointestinal tract, delay gastric emptying, and enhance satiety and fullness. Consequently, appetite and overall energy intake are reduced, supporting the continued interest in glucomannan as a potential functional component in obesity management [68]. KGM has been reported to attenuate elevations in blood cholesterol and glucose levels and suppress hepatic cholesterol synthesis. KGM also binds to bile acids in the gastrointestinal tract, thereby increasing its fecal excretion [70]. Because bile acids are synthesized from cholesterol in the liver, increased excretion necessitates greater hepatic cholesterol utilization for bile acid synthesis. This reduces the cholesterol pool available for other functions, including LDL-C formation, ultimately contributing to lipid-lowering effects [71]. During intestinal absorption, the gel-forming properties of KGM reduce the absorption of dietary cholesterol. Increased intestinal viscosity slows micelle formation between cholesterol and bile acids, thereby decreasing cholesterol uptake by enterocytes and ultimately reducing the amount of cholesterol entering circulation [72]. Recent evidence suggests that, in addition to traditional mechanisms, KGM supplementation promotes favorable alterations in the gut microbiota composition. Specifically, KGM modulates obesity-associated microbial communities to support metabolic health [73]. The gut microbiota is essential for host metabolic homeostasis. Specific taxa, such as *Bacteroidetes* and *Akkermansia muciniphila*, regulate the production of short-chain fatty acids (SCFAs), including butyrate and propionate. SCFAs are closely associated with lipid metabolism and insulin sensitivity [74,75]. In particular, propionate inhibits hepatic cholesterol synthesis [76]. Moreover, SCFAs activate AMPK, thereby promoting fatty acid oxidation and reducing lipid accumulation [77].

#### 3.3.2. Clinical Studies

After an 8-week intervention in patients with type 2 diabetes mellitus, KGM- and inulin-fortified yogurt significantly improved insulin sensitivity and lipid indices. In contrast, FGB and HbA1c levels showed no significant intergroup differences, and body weight and WC decreased similarly in both groups [32]. In contrast, a 14-day RCT in overweight adults showed no significant effects of KGM-containing jelly candy on body weight, BMI, or lipid or glycemic indices. Nonetheless, the WC and hunger intensity decreased, possibly because of the short intervention period and low KGM content [33]. A meta-analysis of six RCTs showed that glucomannan intake produced a significantly greater reduction in body weight than control, with an average weight loss of 1.34 kg observed in studies of ≤8 weeks [34]. Similarly, a meta-analysis by Bessell et al. (2021) confirmed a weight loss effect versus placebo, although the clinical significance was limited and between-study heterogeneity was high [35]. A prospective pre–post intervention study involving 136 overweight and obese adults reported that combined supplementation with glucomannan and *Garcinia cambogia* significantly reduced body weight, body fat, and visceral fat, with concurrent improvements in key metabolic parameters including total cholesterol, triglycerides, and FBG [78]. A recent systematic review by Ghosh et al. (2025) found that glucomannan supplementation (3–5 g/day) was associated with reductions in body weight, BMI, and WC, as well as changes in gut microbiota composition, increased SCFA production, and reduced inflammatory markers [36]. A meta-analysis of lipid profiles showed reductions in total cholesterol, LDL-C, Apo-B, and HDL-C, whereas TG and some lipoprotein ratios showed no consistent effects, and the between-study heterogeneity was high [37]. According to a review by Wharton et al. [79], evidence regarding the weight loss effects of glucomannan is largely inconsistent. Some studies reported significant weight loss, whereas others showed no differences compared with a placebo. In particular, high-dose, long-term studies using PGX formulations have demonstrated weight loss effects, although many studies were limited by a lack of control groups or design-related methodological limitations [79]. Evidence suggests that glucomannan has been investigated at doses of 3–5 g/day and may be associated with small short-term reductions in body weight in some trials. However, findings are inconsistent, between-study heterogeneity is substantial, and the clinical relevance of these effects remains uncertain.

#### 3.3.3. Safety and Adverse Effects

Bessell et al. (2021) reported that glucomannan was generally well-tolerated, with mild, transient, and manageable gastrointestinal adverse effects through increased water intake or gradual dose escalation [35]. Ghosh et al. [36] and Wharton et al. [79] similarly reported good tolerability in adults at daily doses of 1.5–5 g, with no serious adverse events. However, a higher incidence of gastrointestinal symptoms, including constipation, diarrhea, and abdominal bloating, has been observed in some studies using multifiber formulations or high-dose preparations, such as PGX. Importantly, most available trials were of short duration, and long-term safety data remain limited. Safety findings should therefore be interpreted in relation to specific formulations, dosages, and study contexts. Caution is advised in individuals with a history of esophageal or gastrointestinal disease, given the viscous and expandable properties of glucomannan. Additionally, combination studies of glucomannan with *Garcinia cambogia* reported no adverse events over 6 months [36,79]. However, attribution of safety to a single ingredient is limited in multi-ingredient interventions. Within the dose ranges and study durations examined in clinical trials, no consistent signals of serious toxicity have been identified. However, these findings do not establish long-term safety, and interpretation should consider formulation, dosage, duration, and study context.

### 3.4. Garcinia Cambogia

#### 3.4.1. Weight Loss Mechanism

The genus *Garcinia* has drawn interest from both academia and industry for its pharmacological effects, including metabolic and weight regulation and modulation of blood lipid and glucose levels. Consequently, numerous *Garcinia*-based functional health foods are currently marketed online for weight loss [80]. *Garcinia cambogia*, an edible fruit consumed in South Asia, has been traditionally used for preservation and flavor enhancement. The biological activity of *Garcinia* species is attributed to their rich content of bioactive compounds, such as phenols, flavonoids, alkaloids, benzophenones, phloroglucinols, xanthones, organic acids, and biphenyls [81]. The key bioactive constituents include garcinol, isogarcin, hydroxycitric acid (HCA), mangostin, and xanthoquimol [82]. In particular, HCA, which is primarily concentrated in the fruit rind, has been among the most extensively investigated anti-obesity compounds in recent decades and constitutes approximately 20–30% of the fruit’s dry weight [82,83]. Accumulating evidence suggests that the anti-obesity effects of HCA result from an interplay between multiple mechanisms, including de novo lipogenesis inhibition and appetite regulation [84,85,86]. HCA competitively inhibits ATP-citrate lyase and reduced acetyl-CoA availability. Given that acetyl-CoA serves as a central precursor for fatty acid, cholesterol, and triacylglycerol synthesis, this inhibition suppresses lipogenesis and cholesterol biosynthesis, particularly under high-carbohydrate conditions, thereby reducing subcutaneous and visceral fat accumulation and promoting weight loss [87,88]. Consequently, dietary carbon is diverted toward hepatic glycogen synthesis, and these metabolic alterations are proposed to signal the central nervous system, increase serotonin levels, and suppressing appetite [82]. Moreover, reduced acetyl-CoA levels lead to lower malonyl-CoA levels, disinhibiting CPT-1 activity, through which long-chain acyl-CoA may act as a mediator of appetite regulation and energy balance [89]. Consistent with these mechanisms, HCA has been reported to confer benefits beyond weight and food intake reduction, including improvements in inflammation, oxidative stress, insulin resistance, and hyperlipidemia [90,91].

#### 3.4.2. Clinical Studies

Recent clinical evidence suggests that the efficacy of *Garcinia cambogia* remains inconsistent. In diet-controlled overweight and obese adults, supplementation with *Garcinia cambogia* extract at 1.5 g/day reduced body weight and BMI in intervention and control groups. However, greater reductions in WC, increases in basal metabolic rate, decreases in serum creatinine with improved clearance, and increases in cognitively restrained eating behaviors were observed in the intervention group [38]. Amini et al. [39] demonstrated a significant reduction in serum leptin levels compared to placebo, particularly in subgroups with at least 50 participants and a mean age of 30 years or older. Despite these findings, the between-study heterogeneity was high, potentially driven by differences in dosage, intervention duration, sex, and metabolic characteristics [39]. A dose–response meta-analysis further reported significant placebo-adjusted reductions in body weight, BMI, percent body fat, and WC. However, non-linear dose–response patterns and substantial heterogeneity were evident for body weight and BMI [41]. Conversely, other systematic reviews and meta-analyses found no statistically significant weight loss effects of *Garcinia cambogia*. These analyses highlighted the heterogeneous composition of the combination products, incomplete reporting of absolute values, and unclear ingredient and dosage information as key limitations, suggesting an elevated risk of bias and reduced confidence in the results. Moderate to high heterogeneity was observed in both single-ingredient and combination product studies [40]. In line with these findings, Andueza et al. [88] reported improvements in lipid and glycemic outcomes in some studies, whereas weight loss effects were significant in 12 studies and absent in 8 studies, indicating limited overall consistency. Such discrepancies may be attributed to methodological factors, including variation in sample size, sex imbalance, heterogeneity of intervention formulations, challenges in isolating the effects of HCA in combination therapies, and potential lifestyle confounders due to differences in dietary and physical activity control [88]. Taken together, current evidence suggests that *Garcinia cambogia* may be associated with modest short-term changes in certain anthropometric or metabolic parameters in some subgroups. However, findings remain inconsistent, heterogeneity is substantial, and methodological limitations reduce confidence in the overall effect. Therefore, sustained weight loss or clinically meaningful benefit cannot be inferred with certainty based on the currently available evidence.

#### 3.4.3. Safety and Adverse Effects

In multiple RCTs administering HCA at doses of up to 2.8 g/day, no serious adverse events were observed [88]. Additionally, intake of *Garcinia cambogia* at 1.5 g/day during the observation period was confirmed to be safe with respect to liver function, with no changes in TGO or TGP levels and no related clinical symptoms observed or reported [38]. However, prospective studies have reported cases of hepatocellular injury with features of acute hepatitis, occurring between 2 weeks and 12 months after initiation of intake, and this liver injury has been associated with the HLA-B*35:01 allele [42]. A review by Maunder et al. (2020) also indicated that the overall pattern of adverse events was similar to that of placebo, although increased gastrointestinal discomfort was observed in the intervention group in a single study [40]. Multi-ingredient formulations, such as Hydroxycuts, have been associated with serious adverse events including liver injury, acute pancreatitis, and serotonin toxicity, although ugh attribution to a single ingredient remains difficult [88]. Additional case reports of hepatotoxicity have also been reported with HCA-only preparations. However, most cases followed a reversible course after discontinuation [41]. Taken together, while RCTs within the investigated dose ranges did not report serious adverse events, safety interpretation should consider formulation type, study duration, and the inherent limitations of causality assessment in case reports.

## 4. Patterns of Consumer Supplement Choice

### 4.1. Conceptualizing Supplement Use Intention: Insurance-like Perception and Preventive Motivation

In this review, supplement use intention refers to the underlying motivational orientation that shapes decisions to initiate or continue supplement consumption, independent of clinically demonstrated efficacy. Rather than being driven solely by evidence-based assessments of benefit and risk, supplement use intentions often emerge from broader cognitive and social processes. Among these, we conceptualize a recurring pattern as an “insurance-like perception,” defined as the belief that supplement use serves as a precautionary strategy to address perceived future health risks, even in the absence of a clear medical need. Previous studies suggest that supplement use is frequently influenced less by established scientific evidence and more by subjective norms, reward-oriented motivations, vague health expectations, and incomplete or selectively interpreted information. One study reported that supplement use increased with stronger beliefs in health benefits and disease prevention, greater exposure to mass media, and higher expectations regarding supplement efficacy, whereas the influence of healthcare professionals was relatively limited [20]. Conner et al. (2001) similarly found that users often believe supplements prevent disease and enhance health despite limited scientific support, and may view supplementation as “the best thing one can do for oneself,” a perception linked to viewing supplements as a form of insurance against potential diet-related diseases [92]. Consumers also rely more on intuitive beliefs than on formal regulatory warnings, a tendency attributed to activated belief systems that filter out regulatory information during assessment and interpretation [19]. Davis et al. (2008) further suggested that supplement use may serve as a compensatory behavior to offset unhealthy lifestyles, functioning as an alternative health behavior selected to counterbalance unhealthy actions [93]. Related studies have described this pattern as a licensing effect, whereby supplement consumption justifies subsequent unhealthy behaviors. In this framework, supplement use operates as a psychological exemption that weakens self-regulation, allowing use to persist based on distorted goals and reduced self-control rather than clearly defined objectives such as weight loss [94,95]. Moreover, ambiguity in usage intentions may be further reinforced when supplements are perceived not as complementary to meals, but as substitutes capable of replacing meal functions. The belief that supplements can provide nutrients in amounts equivalent to those obtained from meals reflects nutritionism, a perspective that reduces the value of food in terms of individual nutrient components. Consequently, distinctions among whole foods, fortified processed foods, and supplements may become blurred, leading to the perception that health can be achieved through the same mechanisms [96].

### 4.2. Structural and Informational Environments Reinforcing Supplement Use Intentions

Use intentions grounded in beliefs, reward-oriented motivations, and insurance-like perceptions are not merely individual cognitive issues, but structural phenomena repeatedly reinforced by the surrounding social and informational environments. One such structural condition is the Internet-centered distribution of supplement-related information, which may play a critical role in the emergence of ambiguous use intentions. When the quality and sources of information are weak, consumers are more likely to make decisions without adequately recognizing the supplementary efficacy or potential risks, thereby increasing the likelihood of distorted judgments. Indeed, studies on dietary supplement users indicate that approximately 90% of respondents search for information prior to use. However, >80% rely primarily on the Internet as their main information source, despite its insufficient quality, and some reports use the Internet as their sole source. In addition, consultations with healthcare professionals were rare, suggesting that this information environment may contribute to consumers’ limited awareness of both supplementary efficacy and potential harms [97]. Moreover, an analysis of 1179 websites evaluating clinical efficacy and safety information revealed that the majority of online herbal content did not sufficiently address essential safety issues, including potential adverse effects and drug interactions. Notably, fewer than 3% of websites referred to scientific evidence [98]. Moreover, ambiguity in use intentions reflects not only a lack of information, but also a broader structural issue in which individuals increasingly lose their role as autonomous decision-makers regarding their own health amid the commercialization of nutrition and health. Churchill and Churchill noted that the commodification of nutrition shifts individuals away from being the ultimate authority over their health and toward viewing healthy diets and health promotion as products to be purchased in the marketplace. In this context, consumers are guided by choices validated through marketing and external expert discourse, whereas health judgments based on personal experience and observations are progressively excluded. Consequently, supplement use is less determined by clearly articulated, self-generated health goals and more by dependence on external commercial and professional authorities, further deepening the structural ambiguity of use intentions [99].

## 5. Conclusions

In this narrative review, we examined recent clinical evidence on selected supplements for which weight loss effects have been proposed. Although these supplements have been discussed as potential options for obesity management, we found that the consistency of findings regarding their efficacy and safety remains limited, and that the current level of scientific evidence is insufficient to provide strong clinical confidence. These findings suggest that the use of such supplements requires careful judgment that extends beyond simple expectations of effectiveness, taking into account safety, formulation, and context of use. In practice, however, supplement use intentions tend to be justified not by clearly defined health goals or evidence-based evaluations, but rather by subjective beliefs, reward-oriented motivations, and insurance-like perceptions. Such intentions may be further reinforced by limited scientific evidence, qualitatively weak information environments, and prevailing commercial discourse. Therefore, consumers should be guided toward purpose-driven choices that comprehensively consider individual weight management goals and the current level of available scientific evidence. Beyond individual decision-making, this review emphasizes that issues surrounding supplement selection and use reflect broader structural limitations within information environments where limited scientific evidence and commercial narratives coexist. Accordingly, there is a need to strengthen consumers’ capacity for critical interpretation of information related to efficacy and safety, while also encouraging researchers to interpret supplement-related evidence within a broader social and informational context. This review was conducted as a narrative and interpretive synthesis to contextualize recent clinical findings within consumer and informational settings. Accordingly, formal certainty grading and quantitative effect-size integration were not performed. Future studies should incorporate structured evidence grading and clearer differentiation of claims by formulation, dosage, duration, and evidence source to more rigorously evaluate efficacy and safety.

## Figures and Tables

**Table 1:** Summary and Results of Studies Examining L-Carnitine Supplementation.

Author (Year)	Study Design	Participants/Duration	Formulation	Dose Range	Main Findings (Usefulness)	Safety/Adverse Effects
Sangouni et al. (2022) [21]	Randomized double-blind placebo-controlled trial	Adults (Women with overweight/obesity and knee osteoarthritis); *n* = 70; 12 weeks	L-carnitine Supplement	1 g/day	LAP ↓; AIP, AC, CRI-II ↔	NR
Pakravanfar et al. (2020) [22]	Double-blind randomized clinical trial	Adults women with PCOS; *n* = 56; 12 weeks	L-carnitine Supplement	1 g/day	WC ↓; weight, BMI, HC, FM, FFM ↔	NR
Talenezhad et al. (2020) [23]	A systematic review and meta-analysis (37 RCTs)	Adults (Overweight/obese; healthy or with comorbidities); 3–48 weeks	L-Carnitine Supplement	0.25–4 g/day	weight, BMI, fat mass ↓ (especially in overweight/obese); effect attenuates over time	Mild AEs reported in some studies
Hamedi-Kalajahi et al. (2025) [24]	Umbrella meta-analysis (7 meta-analyses; 23 RCTs)	Adults (healthy and diseased; mixed weight status); 2–52 weeks	L-Carnitine Supplement	0.15–4 g/day	Weight ↓; BMI ↓ (substantial heterogeneity); WC ↓	NR
Zamani et al. (2023) [25]	A systematic review and dose–response meta-analysis (41 RCTs)	Adults (diabetic/non-diabetic; overweight/obese); 2–52 weeks	L-Carnitine Supplement	0.25–4 g/day	FBG ↓; HbA1c ↓; HOMA-IR ↓; insulin ↔ (overall); high heterogeneity; effects in subgroups (FBG ≥ 100 mg/dL, ≥12 week, ≥2 g/day, obesity, diabetes)	GI symptoms, musculoskeletal pain, nausea, edema reported in some RCTs

RCTs, randomized controlled trial; NR, Not Reported; AEs, Adverse effects; BMI, Body Mass Index; GI, Gastrointestinal; WC, waist circumference; FBG, Fasting Blood Glucose; HbA1c, glycated hemoglobin; LAP, lipid accumulation product; AIP, atherogenic index of plasma; AC, atherogenic coefficient; CRI-II, Castelli risk index II; PCOS, polycystic ovary syndrome; HOMA-IR, homeostasis model assessment of insulin resistance; HC, hip circumference; FM, fat mass; FFM, fat-free mass; ↓ decrease; ↔ no significant change.

**Table 2:** Summary and Results of Studies Examining Green Tea Supplementation.

Author (Year)	Study Design	Participants/Duration	Formulation	Dose Range	Main Findings (Usefulness)	Safety/Adverse Effects
Farhadian et al. (2020) [26]	Double-blind randomized clinical trial	Adults Women with PCOS; *n* = 45; 12 weeks	GT capsule	three times daily (1.5 g/day)	Weight, BMI, WC HC ↓; WHR ↔	NR
Hadi et al. (2020) [27]	Randomized, double-blind, placebo-controlled trial	Adult with T2DM; *n* = 44; 8 weeks	EGCG capsule	twice daily (300 mg/day)	Weight, BMI ↓; FBS ↓; hs-CRP ↓; insulin, HOMA-IR, QUICKI ↔	NR
Lin et al. (2020) [28]	A systematic review and dose–response meta-analysis (26 RCTs)	Adults (overweight/obese and metabolic conditions); *n* = 1344; 2–20 weeks	GT/ GTE (beverage, capsules)	0.1–20.0 g/day (most effective range < 0.8 g/day)	Weight, BMI ↓; WC ↔; nonlinear dose–response; moderate heterogeneity	Limited safety data; mostly mild AEs reported
Xiao et al. (2024) [29]	A systematic review and meta-analysis (24 RCTs)	Adults (metabolic syndrome and/or obesity); *n* = 1672; 6–24 weeks	GT/GTE (various types)	0.3–1.0 g/day (maximum catechin dose: 0.928 g/day)	Weight, BMI ↓; lipids ↓; WC/WHR ↔; metabolic markers ↔; moderate–high heterogeneity	Limited safety data; AEs not analyzed
Macêdo et al. (2022) [30]	A systematic review	Obese; 4–20 weeks	GTE/catechins (EGCG); capsules or beverages	EGCG 0.188–0.300 g/day; total catechins up to 0.928 g/day	lipids ↓ (TG, TC, LDL-C ↓; HDL-C ↑ in select studies)	Limited safety data
Rondanelli et al. (2022) [31]	Randomized double-blind placebo-controlled trial	Overweight post-menopausal and class I obese sedentary women; *n* = 28; 60 days	Green tea extract (*Camellia sinensis*; phytosome formulation)	Greenselect^®^ Phytosome^®^ 300 mg/day (catechins ≥19–25%; EGCG ≥ 13%)	RQ ↓; REE, %FAT oxidation ↑; insulin, HOMA-IR ↓; WC, VAT ↓; CRP ↓	No safety/ADs data reported

RCTs, randomized controlled trials; GT, Green tea; GTE, Green tea extract; BMI, body mass index; WC, waist circumference; AEs, Adverse effects; TGs, triglycerides; TC, total cholesterol; LDL-C, low-density lipoprotein cholesterol; HDL-C, high-density lipoprotein cholesterol; WHR, waist-to-hip ratio; NR, Not reported; EGCG, Epigallocatechin-3-gallate; RQ, respiratory quotient; REE, resting energy expenditure; %FAT oxidation, percentage of fat oxidation; HOMA-IR, Homeostasis Model Assessment of Insulin Resistance; VAT, visceral adipose tissue; CRP, C-reactive protein; ↑ increase; ↓ decrease; ↔ no significant change.

**Table 3:** Summary and Results of Studies Examining Glucomannan Supplementation.

Author (Year)	Study Design	Participants/Duration	Formulation	Dose Range	Main Findings (Usefulness)	Safety/Adverse Effects
Dehzad et al. (2025) [32]	Randomized, double-blind, placebo-controlled trial	Adults with T2DM; *n* = 73; 8 weeks	Low-fat yogurt (150 g/day) enriched with KMG/inulin	GM 1.5 g/inulin 1.5 g	insulin ↓; HOMA-IR ↓, QUICKI ↑; TC, TG ↓; TG/HDL-C, TC/HDL-C ↓; body weight, WC, fat mass ↓	No AEs reported
Fernandes et al. (2023) [33]	Randomized, double-blind, placebo-controlled trial	Adult Overweight women; *n* = 42; 14 days	Gummies candy with KMG 250 mg/candy	2 candies/day (500 mg/day)	WC, hunger intensity ↓; weight, BMI ↔; glucose, TC, TG, HDL, LDL, VLDL ↔; TBARS, GSH, NPSH ↔	No AEs reported
Mohammadpour et al. (2020) [34]	A systematic review and meta-analysis (6 RCTs)	Overweight/obesity; *n* = 225; 5–12 weeks	GM (capsule/powder)	1.2–3.9 g/day	Weight ↓; statistically significant but small effect; high heterogeneity	Mostly mild GI issues; overall safe.
Bessell et al. (2021) [35]	A systematic review and meta-analysis (7 RCTs)	Overweight/obesity; *n* = 539; 5–52 weeks	GM (single-ingredient/combined fiber; capsule/powder)	0.42–6.0 g/day	Weight ↓; statistically significant but clinically insignificant; high heterogeneity	Mostly mild, transient GI issues; no serious reported.
Ghosh et al. (2025) [36]	A systematic review (10 RCTs + 9 reviews)	Overweight/obese adults; 6–24 weeks	GM (capsule/powder)	3–5 g/day	BMI, weight, WC ↓; TG, LDL, glucose ↓; gut microbiota ↑	Mild, transient GI issues
Musazadeh et al. (2024) [37]	GRADE-assessed systematic review and meta-analysis (11 RCTs)	Adults; *n* = 334; 3–12 weeks	GM (capsule/powder/ flour/food-based)	1–15 g/day	TC, LDL-C, Apo-B ↓; TG, Apo-A1, Apo-B/Apo-A1 ratio, LDL-C/HDL-C ratio ↔; statistically significant but clinically insignificant effects; high heterogeneity.	Well tolerated; mostly mild GI issues

RCTs, randomized controlled trials; AEs, Adverse effects; KMG, konjac glucomannan; GM, glucomannan; TGs, triglycerides; WC, waist circumference; TC, total cholesterol; Apo-B, apolipoprotein B; Apo-A1, apolipoprotein A1; LDL-C, low-density lipoprotein cholesterol; HDL-C, high-density lipoprotein cholesterol; VLDL, very low-density lipoprotein; GI, gastrointestinal; T2DM, type 2 diabetes mellitus; HOMA-IR, Homeostasis Model Assessment of Insulin Resistance; QUICKI, Quantitative Insulin Sensitivity Check Index; BMI, body mass index; TBARSs, thiobarbituric acid reactive substances; GSH, reduced glutathione; NPSH, non-protein thiol; ↑ increase; ↓ decrease; ↔ no significant change.

**Table 4:** Summary and Results of Studies Examining *Garcinia cambogia* Supplementation.

Author (Year)	Study Design	Participants/Duration	Formulation	Dose Range	Main Findings (Usefulness)	Safety/Adverse Effects
Sant’Ana et al. (2025) [38]	Controlled clinical trial	Adults (overweight and obese women); *n* = 34; 4 weeks	GC extract	three times daily (1.5 g/day; HCA, 750 mg/day)	weight, BMI ↓; WC ↓; BMR ↑; lipid profile, glucose ↔; body fat %, FFM ↔; CR ↑	No AEs reported at 1.5 g/day
Amini et al. (2024) [39]	A systematic review and meta-analysis (8 RCTs)	Adults (healthy/overweight/obese; NAFLD); *n* = 330; 11 days–10 weeks	HCA (supplement)	HCA, 1.4–3.0 g/day (median ≈ 2.9 g/day)	Serum leptin ↓; high heterogeneity	Mostly mild GI issues; rare hepatotoxicity reports
Maunder et al. (2020) [40]	A systematic review and meta-analysis (11 RCTs)	Adults (overweight/obese); *n* = 967; 8–17 weeks	GC extract (single/combination products)	HCA, 1.2–2.8 g/day	Weight loss not significant; moderate–high heterogeneity.	No AEs difference vs. placebo; occasional GI discomfort
Golzarand et al. (2020) [41]	A systematic review and dose–response meta-analysis (8 RCTs)	Adults overweight and obese; *n* = 530; 8–12 weeks	GC supplement (single-herb products)	GC, 0.2–4.7 g/day	Weight, BMI, PFM, WC ↓; nonlinear dose–response for weight and BMI; substantial heterogeneity	Limited safety data; potential hepatotoxicity concerns; liver enzyme monitoring suggested
Vuppalanchi et al. (2022) [42]	Prospective observational study (DILIN cohort analysis)	Adolescents and adults (17–54 y); *n* = 22; Latency, 13–223 days (≈2 weeks–12 months)	GC extract (HCA-containing); single or combination products	NR	Not assessed (efficacy not evaluated)	Moderate to severe hepatocellular liver injury with jaundice reported; association with HLA-B*35:01 observed

NR, Not Reported; BMR, basal metabolic rate; FFM, fat-free mass; CR, cognitive restraint; NAFLD, Non-Alcoholic Fatty Liver Disease; HCA, (−)-hydroxycitric acid; GI, Gastrointestinal; AEs, Adverse effects; GC, *Garcinia cambogia*; BMI, body mass index; PFM, percent fat mass; WC, waist circumference; ↑ increase; ↓ decrease; ↔ no significant change.

## Data Availability

No new data were created or analyzed in this study. Data sharing is not applicable to this article.

## Abbreviations

The following abbreviations are used in this manuscript:

BMI	Body mass index
EGCG	Epigallocatechin gallate
RCT	Randomized controlled trial
WC	Waist circumference
LDL-C	Low-density lipoprotein cholesterol
HDL-C	High-density lipoprotein cholesterol
TC	Total cholesterol
TGs	Triglycerides
FBG	Fasting blood glucose
HbA1c	Glycated hemoglobin
HOMA-IR	Homeostasis model assessment of insulin resistance
AMPK	AMP-activated protein kinases
WHR	Waist-to-hip ratio
CRP	C-reactive protein
KGM	Konjac glucomannan
SCFAs	Short-chain fatty acids
HCA	Hydroxycitric acid
